# Electricity energy dataset “BanE-16”: Analysis of peak energy demand with environmental variables for machine learning forecasting

**DOI:** 10.1016/j.dib.2023.109967

**Published:** 2023-12-18

**Authors:** Imrus Salehin, S.M. Noman, Mohammad Mahedy Hasan

**Affiliations:** a Department of Computer Science and Engineering, Daffodil International University, Dhaka 1216, Bangladesh; b Department of Electrical and Electronic Engineering, Daffodil International University, Dhaka 1216, Bangladesh; c Faculty of Computer Science and Engineering, Frankfurt University of Applied Sciences, Frankfurt 60318, Germany; d Faculty of Physics and Electrical Engineering, University of Bremen, Bremen 28359, Germany; e Department of Computer Engineering, Dongseo University, 47 Jurye-ro, Sasang-gu, Busan 47011, the Republic of Korea

**Keywords:** Energy, Electricity, Forecasting, AI, Machine Learning

## Abstract

The “BanE-16” dataset is a comprehensive repository integrating electricity grid dynamics with meteorological variables for machine learning-based energy forecasting. Featuring peak energy demand, environmental factors (temperature, wind speed, atmospheric pressure), and electricity generation statistics, this dataset enables intricate analysis of weather-energy correlations. Its multidimensional nature facilitates predictive modeling, exploring intricate dependencies, and optimizing energy infrastructure. Leveraging machine learning methodologies, this dataset stands as a catalyst for innovative forecasting models and informed decision-making in energy management. Its diverse variables offer a holistic perspective, empowering researchers to delve into nuanced interrelationships, paving the way for sustainable energy planning and predictive analytics in dynamic energy ecosystems. Its multivariate nature empowers sophisticated machine-learning models, enabling precise energy forecasts and infrastructure optimizations. Researchers leveraging this dataset unlock the potential to delve deeper into intricate weather-energy relationships, driving advancements in predictive analytics for sustainable energy management. The integration of diverse variables lays the groundwork for innovative methodologies, steering the trajectory of informed decision-making in dynamic energy landscapes.

Specifications Table

**Table:** 

Subject	Data Mining, Statistical Analysis, Electrical Engineering, Energy, AI
Specific subject area	Numerical Data Analysis for Peak Energy Demand Forecasting in the Electricity Energy using Machine Learning Approach.
Data format	Raw, Filtered.
Type of data	Filtered measurement data is stored as .csv files.
Data collection	The data collection process involved employing specialized devices to capture temperature and wind data at various locations. These devices were strategically placed to record accurate and localized measurements across different points of interest. Simultaneously, the electrical data was collected from authoritative sources, predominantly governmental and regulatory organizations. These organizations regularly publish detailed datasets encompassing various aspects of electrical information, including consumption patterns, grid performance, and other relevant metrics. Careful attention was paid to ensuring the accuracy and reliability of the collected data, employing quality control measures to validate and cross-reference information from different sources. The comprehensive approach in data collection aimed to provide a holistic and reliable dataset for subsequent analysis and forecasting of peak energy demand within electricity power.
Data source location	Department of Computer Science and Engineering and Department of Electrical and Electronics Engineering, Daffodil International University, Dhaka, Bangladesh, and Bangladesh Power Development Board (BPDB).
Data accessibility	Repository name: Mendeley data Data identification number: doi: 10.17632/3brbjpt39s.2 Direct URL to data: https://data.mendeley.com/datasets/3brbjpt39s/2

## 1 Value of the Data

• These datasets hold immense value as they provide crucial information for accurately predicting energy demand. Wind speed and temperature data enable precise forecasting by indicating how weather conditions impact energy consumption. The maximum energy generation data aids in grid management, ensuring a stable supply during peak periods.
• Other researchers can use these datasets to improve their forecasting models, validate their findings, or explore new insights related to energy demand. The data can aid in refining existing models, studying climate impacts, designing better energy policies, and serving as educational tools for aspiring researchers. Overall, these datasets provide a valuable foundation for advancing research in energy demand forecasting and renewable energy integration.
• Given the dataset's frequent measurements and diverse environmental variables it proves valuable for researchers exploring rainfall prediction, as well as estimating annual electricity generation using data mining techniques for modeling, estimation, and prediction purposes.
• This dataset, encompassing operational and reference performance measurements, serves as an excellent resource for researchers investigating the correlation between machine learning and electrical field integration. It presents an opportunity for developing automation systems within the electrical domain.
• This dataset amalgamating real-time operational metrics and historical performance benchmarks, provides an invaluable resource for researchers delving into the convergence of machine learning and electrical systems. It facilitates the development of sophisticated predictive models and intelligent algorithms aimed at enhancing the reliability and efficiency of electrical infrastructure in dynamic operational environments.

## 2 Background

The primary background behind collecting this dataset revolves around capturing essential information regarding energy demand and consumption patterns. This dataset acquisition was driven by the goal of comprehensively understanding and analyzing the dynamics of energy usage, facilitating the development of predictive models, and exploring potential advancements in energy management systems. Weather data collection for energy demand research serves as a crucial component in understanding the intricate relationship between weather patterns and energy consumption. Gathering this information is pivotal for accurately predicting energy demands, as weather elements like temperature, humidity, wind speed, and precipitation significantly influence energy usage patterns. The collection of weather data supports the development of models that can anticipate energy demands based on varying climatic conditions, aiding in efficient energy distribution and management. The aim of collecting energy demand, consumption data and environment data lies in integrating Electrical and AI domains. Our goal is to fuse these energy-related fields with AI-driven automation. Given the current global trajectory favoring AI advancements, we intend to harmonize these fields to develop innovative solutions for energy automation empowered by AI technologies.

## 3 Data Description

We have gathered daily peak energy consumption data from the Bangladesh Power Development Board (BPDB) spanning January 2018 to April 2023, encompassing seven distinct zones. Our focus centers on peak energy consumption and the highest daily energy demand while exploring related meteorological factors such as temperature (minimum, maximum, and average readings), wind patterns, humidity levels, cloud cover, and precipitation. Table 1 shows all data columns with descriptions.

**Table 1:** An outline of the variables measured in the BanE-16 dataset.

Name of data column	Description
Month	Represents the month of the recorded data.
Year	Indicates the year of the recorded data.
Temp2(c)	Temperature at a certain location using the sensor. (Celsius °C)
Temp2_max(c)	Maximum temperature recorded within a specific period. (Celsius °C)
Temp2_min(c)	Minimum temperature recorded within a specific period. (Celsius °C)
Temp2_ave(c)	Average temperature recorded within a specific period. (Celsius °C)
Suface_pressure(pa)	Atmospheric pressure at the surface level.
Wind_speed50_max(m/s)	Maximum wind speed recorded at 50 m above ground level.
Wind_speed50_min(m/s)	Minimum wind speed recorded at 50 m above ground level.
Wind_speed50_ave(m/s)	The average wind speed was recorded at 50 m above ground level.
Prectotcorr	Precipitation correlation or precipitation-related measurement.
Total_demand(mw)	Total electricity demand is measured in megawatts.
Max_generation(mw)	Maximum electricity generation recorded in megawatts.

For energy demand and consumption data, we gather information from the BPDM public dataset available on the government's website. Collecting raw data for parameters like daily energy demand, temperature variations, wind patterns, humidity levels, cloud cover, and precipitation involves deploying specialized devices and sensors tailored for each parameter. For instance, energy meters and smart meters are integrated into the electricity grid to record daily energy consumption. Weather station (Daffodil University Lab) equipped with diverse sensors, such as thermocouples for temperature, anemometers for wind patterns, and hygrometers for humidity levels, are strategically positioned to capture localized weather data. These stations also employ sky imagers or ceilometers to assess cloud cover and rain gauges to measure precipitation. These devices collectively provide a comprehensive dataset reflecting the dynamic environmental conditions. Regular calibration and positioning of these devices ensure the accuracy and reliability of the collected raw data, forming the basis for detailed analysis and insights into weather patterns, energy demand fluctuations, and their correlations over time. This initiative of data gathering process focuses on global warming reduction and sustainable management, accumulated by a device known as the Netatmo weather station in Innovation LAB.

To ensure consistency and accuracy, we have adopted standardized data collection [1] methodologies uniformly applied across all different zones (inside the university area). Our primary objective involves studying the correlation between peak energy usage and various weather parameters [2]. This analysis aims to uncover insights into how weather conditions impact energy demand across Bangladesh.

This dataset encompasses a wide array of weather-related [3] variables alongside crucial energy metrics, enabling a multifaceted exploration into the intersection of weather patterns and energy dynamics. With detailed records of temperature variations, atmospheric pressure, wind speeds at different levels, and potential precipitation correlations, researchers can unveil intricate relationships between weather conditions and energy demand or generation patterns. The temporal aspect provided by the month and year columns allows for time-based analysis, facilitating trend identification and seasonal impacts on energy consumption and generation. These combined factors provide a robust foundation for comprehensive studies delving into the influence of weather phenomena on electricity demand, generation capacities, and the broader implications for energy infrastructure planning and resilience. Researchers can utilize this dataset for their analyses.

## 4 Experimental Design, Materials and Methods

**Fig. 1:**
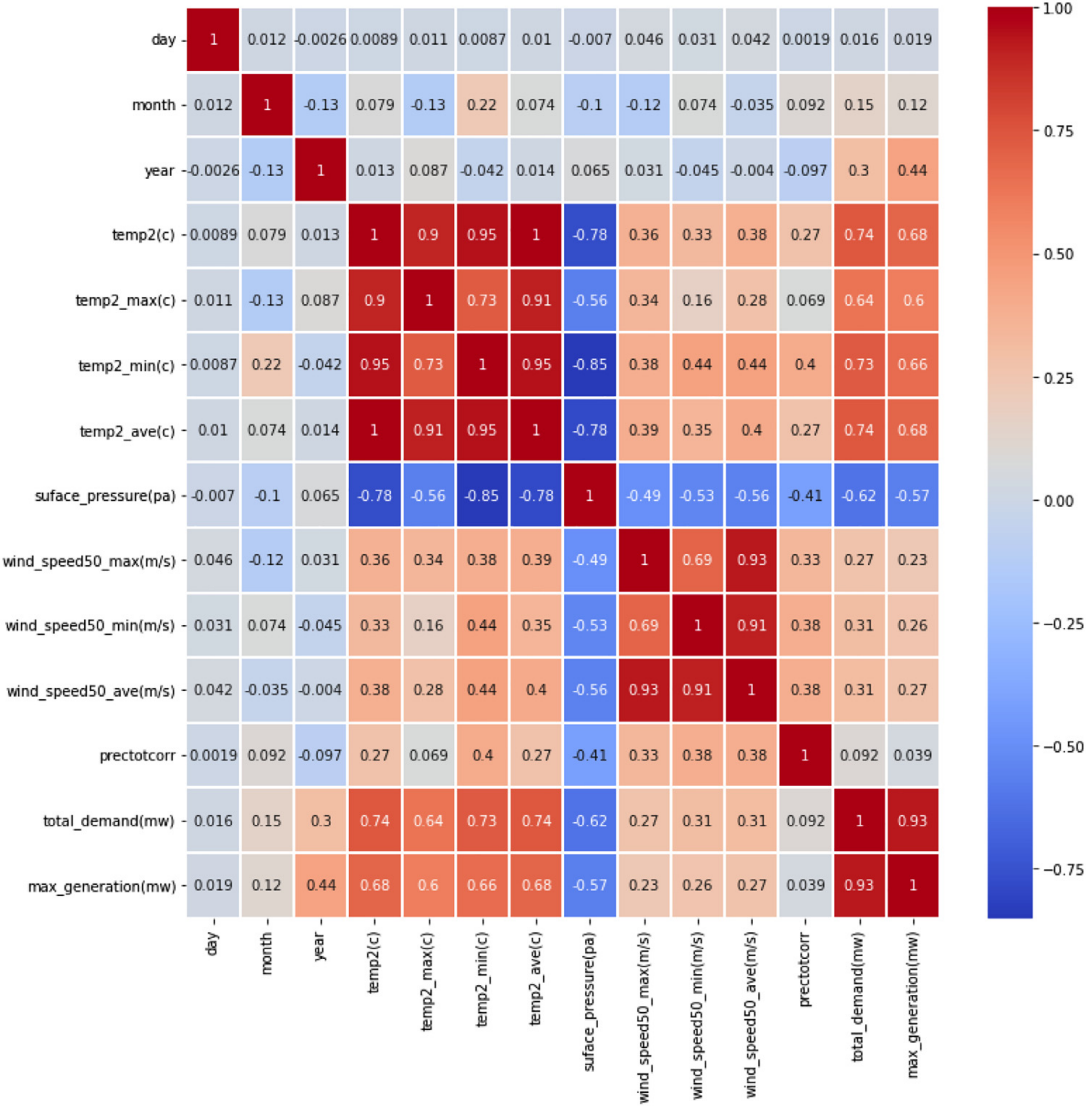
Heat map displaying the correlations between the dataset variables.

The data analysis includes various factors: peak energy consumption, temperature (minimum, maximum, and average), wind speed, humidity levels, cloud cover, and rainfall. Each variable is visualized in the diagram, showcasing their interconnectedness. Employing correlation analysis enables the identification of potential dependencies and patterns within the dataset, shedding light on how meteorological variables might affect peak energy consumption in Bangladesh. Fig. 1 illustrates the results of the correlation analysis conducted on the primary data variables.

**Fig. 2:**
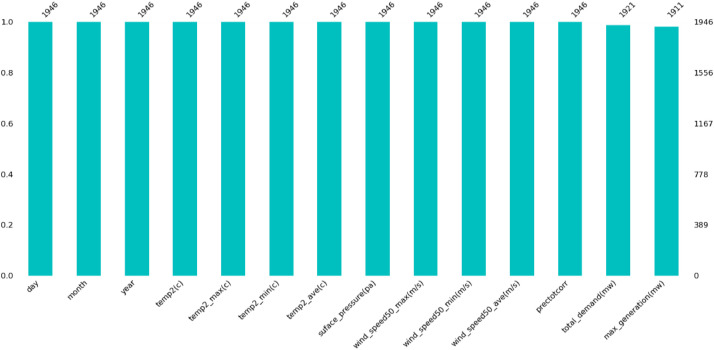
Showcases the absence of values in demand and generation.

Fig. 2 provides a detailed illustration of the missing data observed in the statistics related to peak energy consumption (demand) and generation. This analysis specifically highlights instances where these pivotal variables exhibit data gaps. Although the proportion of missing data is relatively small, this image is crucial for pinpointing any inconsistencies within the dataset. Such visual representation significantly contributes to addressing concerns regarding data completeness and helps ensure a more comprehensive dataset evaluation.

A histogram represents the distribution of data by dividing it into intervals or bins and displaying the frequency of occurrences within each bin as bars. In the context of this dataset, a histogram could visualize various aspects:

• **Energy Demand Distribution**: A histogram can display the frequency or distribution of energy demand values, showcasing how often different demand levels occur within the dataset.
• **Temperature Range**: It can illustrate the distribution of temperatures recorded, indicating how frequently specific temperature ranges, like minimum, maximum, or average temperatures, occur.
• **Wind Speed Distribution**: Showcasing the frequency of different wind speed ranges recorded within the dataset, providing insights into prevailing wind conditions.
• **Other Variables**: Histograms can also represent the distribution of other parameters like humidity, cloud cover, or rainfall, elucidating their occurrence patterns.

**Fig. 3:**
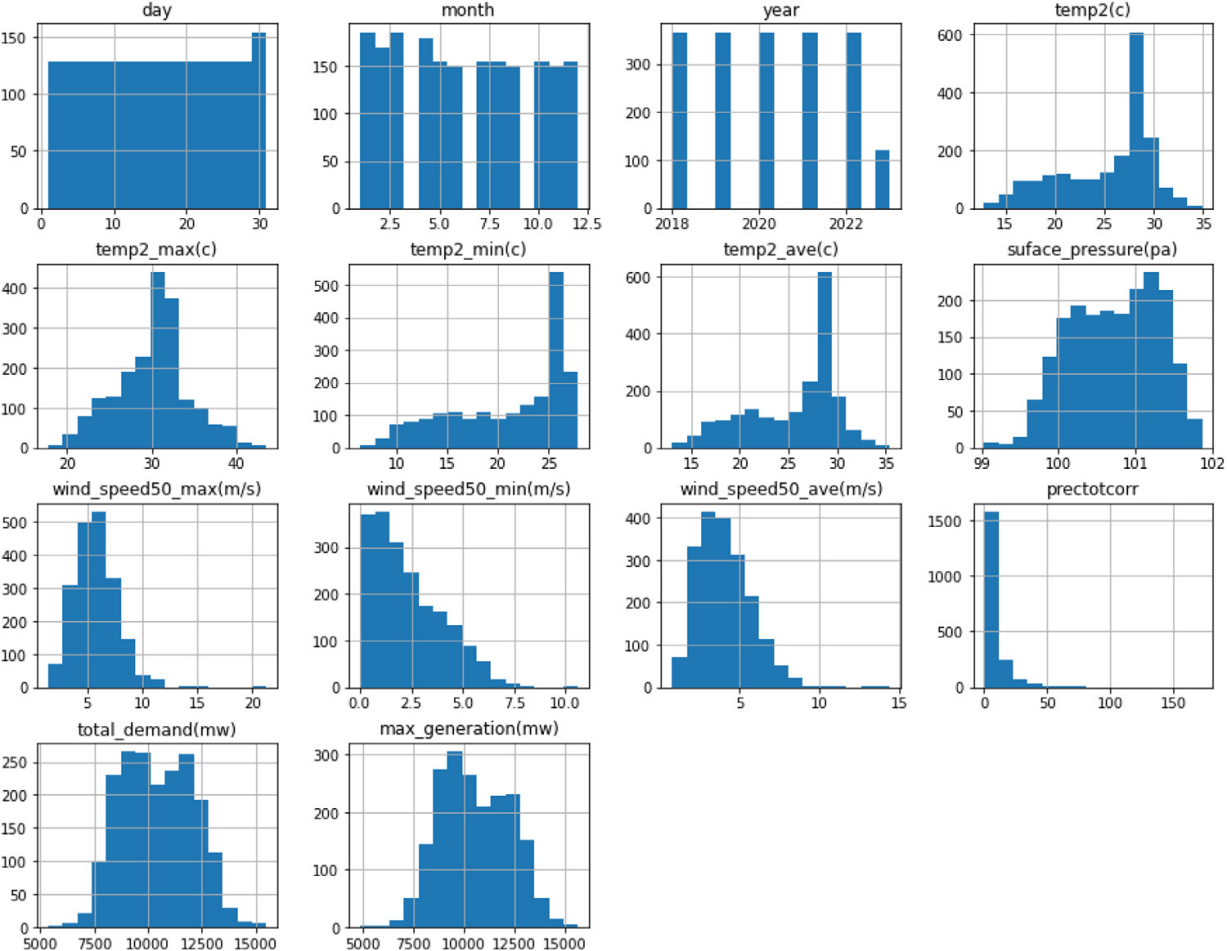
Distribution of energy demand levels across days, months, and years, considering wind, temperature, pressure, demand, and generation variables.

In essence, a histogram in this dataset could visually represent the occurrence frequency of specific values or ranges within different variables, offering insights into their distribution patterns and frequencies within the dataset. In Fig. 3, we illustrate the distribution of energy demand and other data variables using a histogram, displaying the frequency of various demand levels within the dataset.

In this dataset, several well-known machine learning algorithms can be employed for prediction system development. This includes Linear Regression, Random Forest, and binary classification algorithms. Additionally, we can utilize artificial neural networks, especially deep learning algorithms such as LSTM (Long Short-Term Memory) [5] and RNN (Recurrent Neural Network), given that this dataset contains time sequence data.

The methods of linear power flow model, data-driven models, Monte Carlo methods and machine learning techniques are utilized to improve the generation of power system data [6]. In our estimation forecasting, we incorporate the Random Forest algorithm from the realm of machine learning to develop accuracy. This data can contribute to future research aimed at understanding the relationship between energy production and environmental impacts, thereby supporting efforts to address climate changes to promote sustainable clean energy. The proposed models comprise four key steps: data collection, data sampling, data preprocessing, and data integration into machine learning techniques. Data analysis intends to enhance sustainable energy management and power system planning in the South Asia region by integrating long-term energy forecasts that consider geographical correlations.

Future research utilizing the BanE-16 dataset holds promise for advancing energy forecasting methodologies. By delving into novel machine learning and deep learning architectures customized for energy prediction, researchers can harness the dataset's attributes to design more precise models. Furthermore, exploring the incorporation of external factors such as socio-economic indicators or geographical data could enhance the accuracy and robustness of forecasting models. Deeper analyses of temporal patterns and regional variations within the dataset offer avenues to uncover nuanced trends, seasonal effects, and geographical disparities in energy demand. Collaborations across disciplines, integrating insights from energy experts, data scientists, and domain specialists, can foster innovative methodologies for more accurate and adaptable forecasting. Moreover, leveraging the dataset to navigate emerging trends like renewable energy integration, smart grid technologies, or demand-side management will aid in developing forward-looking forecasting models aligned with evolving energy landscapes.

The intricate interplay between weather elements and energy dynamics unveils a complex web of dependencies within the weather-energy domain. Specific correlations highlight the direct impact of weather patterns on peak energy demand and generation. For instance, temperature fluctuations significantly influence energy consumption, particularly during extreme weather conditions. Similarly, the relationship between wind speed and renewable energy generation showcases how natural elements directly affect energy supply. Exploring the correlation between humidity levels and cooling requirements underscores the subtle nuances in energy usage patterns. Moreover, seasonal variations play a pivotal role, delineating the direct influence of weather on distinct energy demands throughout the year.

In conclusion, the dataset offers a comprehensive collection of variables encompassing energy demand, weather parameters, and generation statistics. Analysis of this dataset provides valuable insights into the interplay between meteorological factors and energy consumption, facilitating informed decision-making for sustainable energy planning and infrastructure development [4].

## Limitations

The dataset discussed in this article presents several limitations that warrant consideration. One notable concern is the potential presence of missing or inconsistent data which can compromise the overall quality and completeness of the dataset. Additionally, the relatively small sample size might restrict the generalizability of findings derived from the analysis. There's also a possibility of inherent biases within the data, stemming from factors like geographic or temporal variations, which could influence interpretations and outcomes. The dataset's scope might lack essential variables crucial for comprehensive analysis, while variations in data collection methods over time might introduce inconsistencies.

## Ethics Statement

The authors have adhered to the ethical standards for Data in Brief publication. They affirm that their work did not entail data collection from human subjects, animal experiments, or social media platforms. Some public data from the Bangladesh Power Development Board (BPDB) has been utilized by us without any conflicts.

## CRediT authorship contribution statement

**Imrus Salehin:** Investigation, Software, Visualization, Writing – original draft, Writing – review & editing. **S.M. Noman:** Conceptualization, Data curation, Methodology, Writing – review & editing. **Mohammad Mahedy Hasan:** Conceptualization, Data curation, Methodology, Writing – review & editing.

## Data Availability

Peak Energy Demand in the Electricity Energy Dataset BanE-16 (Original data) (Mendeley Data)
